# Long-lasting renewable antibacterial porous polymeric coatings enable titanium biomaterials to prevent and treat peri-implant infection

**DOI:** 10.1038/s41467-021-23069-0

**Published:** 2021-06-03

**Authors:** Shuyi Wu, Jianmeng Xu, Leiyan Zou, Shulu Luo, Run Yao, Bingna Zheng, Guobin Liang, Dingcai Wu, Yan Li

**Affiliations:** 1 Department of Prosthodontics, Hospital of Stomatology, Guanghua School of Stomatology, Guangdong Provincial Key Laboratory of Stomatology, Sun Yat-sen University, Guangzhou, 510055 P. R. China; 2 Materials Science Institute, PCFM Lab and GDHPRC Lab, School of Chemistry, Sun Yat-sen University, Guangzhou, 510275 P. R. China

**Keywords:** Antimicrobials, Inorganic chemistry, Biomaterials, Implants

## Abstract

Peri-implant infection is one of the biggest threats to the success of dental implant. Existing coatings on titanium surfaces exhibit rapid decrease in antibacterial efficacy, which is difficult to promisingly prevent peri-implant infection. Herein, we report an N-halamine polymeric coating on titanium surface that simultaneously has long-lasting renewable antibacterial efficacy with good stability and biocompatibility. Our coating is powerfully biocidal against both main pathogenic bacteria of peri-implant infection and complex bacteria from peri-implantitis patients. More importantly, its antibacterial efficacy can persist for a long term (e.g., 12~16 weeks) in vitro, in animal model, and even in human oral cavity, which generally covers the whole formation process of osseointegrated interface. Furthermore, after consumption, it can regain its antibacterial ability by facile rechlorination, highlighting a valuable concept of renewable antibacterial coating in dental implant. These findings indicate an appealing application prospect for prevention and treatment of peri-implant infection.

## Abstract (web summary)

Infection is a major problem for dental implants with current antibacterial coatings losing efficacy quickly. Here, the authors report on the N-halamine polymeric coating of titanium implants to create a long-lasting renewable antibacterial layer and demonstrate application in vivo.

## Introduction

Dental implant is currently the preferred alternative for restoring the function and aesthetic morphology of lost teeth^1^. According to a report from the World Health Organization, ~10 million people require restoration of lost teeth each year. The global dental implant market was valued at US$3.77 billion in 2016 and has consistently grown since then, showing that currently there are high demands for dental implants^2^. Because of good mechanical properties, biocompatibility and corrosion resistance^3,4^, titanium-based metal materials are the most commonly used materials for dental implants. However, titanium materials not only have good biocompatibility with host cells but also with bacteria, so bacterial infections frequently happen during dental implantation, causing big challenges.

**Fig. 1:**
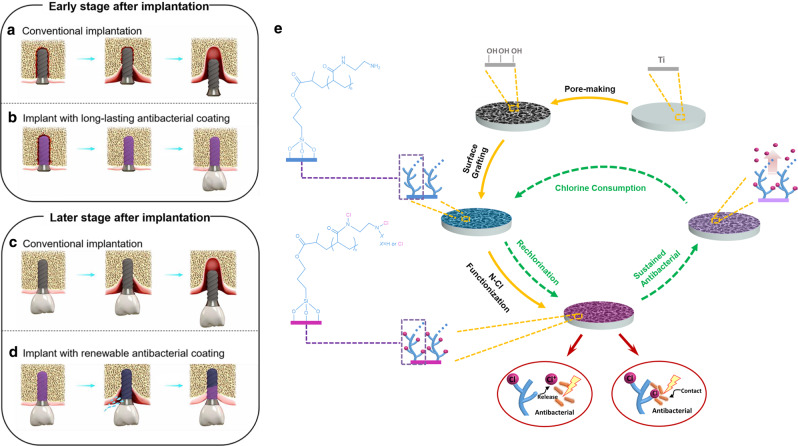
Schematic illustration about how the porous N-halamine polymeric coating on the titanium surface prevents and treats peri-implant infection. **a** Early loss of the implant is easily caused by bacterial invasion prior to the formation of osseointegrated interface. **b** Long-lasting antibacterial property of N-halamine polymeric coating guarantees an effective antibacterial protection to prevent the early peri-implant infection and improve the success rate of dental implantation in the early stage. **c** Once the peri-implantitis occurs at the later stage, it is very difficult to reverse, ultimately resulting in the loss of the dental implant. **d** After being consumed, if peri-implantitis occurs, the antibacterial component of N-halamine polymeric coating can be simply regenerated by peri-implant irrigation, and the exposed implant surface will regain antibacterial property to resist bacterial invasion and control peri-implantitis. **e** Chemical synthesis process and antibacterial mechanism of the porous N-halamine polymeric coating on the titanium surface.

The tissue around the implant is different from natural periodontal tissue and its ability to defend against bacterial invasion is relatively weak^5,6^. This is especially the case in an unhealthy microecological environment, including environments containing periodontitis, maxillofacial defects and infection of the alveolar socket, which are more likely to occur in patients with peri-implant infection. Actually, peri-implant infection may arise at all time points, starting from the moment at which implants are inserted. However, the initial 4 weeks after implantation are the peak period of infection^7^, because osseointegrated interface has not yet formed and the anti-infection ability of the interface is quite weak, probably leading to early loss of the implant prior to the restoration of supra-construction (Fig. 1a). Therefore, the long-lasting antibacterial property of the implant surface to prevent infection until the osseointegrated interface completely forms is in urgent need (Fig. 1b).

Progress has been made in reducing bacterial adhesion and inhibiting biofilm formation through antibacterial modification of the surface of the implant materials to prevent infection in the early stage after implantation^8–10^. However, antibacterial coatings on titanium surfaces, which are created through non-covalent adsorption (e.g. hydrogen bonding and electrostatic interactions), are less firm, and the antibacterial components may quickly desorb from the surface^11,12^. On the other hand, traditional chemical coating methods, which often involve the application of antibiotics and antibacterial peptides to a modified titanium surface, show a rapid decrease in antibacterial efficacy over time^13^. As a result, all of the above methods are very difficult to achieve the desired long-lasting antibacterial goal in Fig. 1b.

Peri-implantitis is peri-implant infection in the later stage after osseointegration, consisting of both soft tissue inflammation and progressive supporting bone loss around an implant beyond biological bone remodelling^14^. The incidence of peri-implantitis is as high as 20–47%^15^, and it is currently a key cause of the failure of the dental implant restoration^16,17^. Nowadays, clinical treatments of peri-implantitis predominantly use instruments or laser debridement combined with local or systemic antibiotics to remove biofilm and control infection^18^. However, the complex surface characteristics of dental implants, irregular bone defect morphology and limited therapeutic approaches tend to decrease the effectiveness of mechanical debridement, and the recurrent use of antibiotics increases the risk of bacterial resistance^19^. Therefore, once peri-implantitis occurs, it is very challenging to reverse^20^, which unavoidably causes continuous destruction of the supporting tissues and eventually leads to the loss of the dental implant (Fig. 1c). On the contrary, if the antibacterial property of the implant surface can be simply regenerated by peri-implant irrigation, the exposed implant surface deriving from bone resorption will regain antibacterial property to resist bacterial invasion by itself. We believe that such a renewable antibacterial implant surface may be able to treat peri-implantitis and stop the progress of supporting bone loss, which eventually avoids the failure of dental implantation (Fig. 1d). However, to the best of our knowledge, there are rare reports about modification with renewable antibacterial property on the surface of dental implant materials.

In this work, we design and construct a porous N-halamine polymeric coating on the titanium surface, which can simultaneously meet the long-lasting and renewable antibacterial demands. The long-lasting antibacterial property of N-halamine polymeric coating fully covers the osseointegration-forming period and even beyond, and thus effectively prevents peri-implant infection in the early stage after implantation and even peri-implantitis before it happens (Fig. 1b). After being consumed, if peri-implantitis happens, the active chlorine in N-halamine coating can be regenerated by simple peri-implant irrigation to regain its antibacterial ability, which can resist both bacteria and biofilm and thus greatly improve the cure rate of peri-implantitis (Fig. 1d). All in all, our well-orchestrated modification shows a very appealing application prospect of both prevention and treatment of peri-implant infection.

## Results

### Preparation and characterization of the porous N-halamine polymeric coating on the titanium surface

**Fig. 2:**
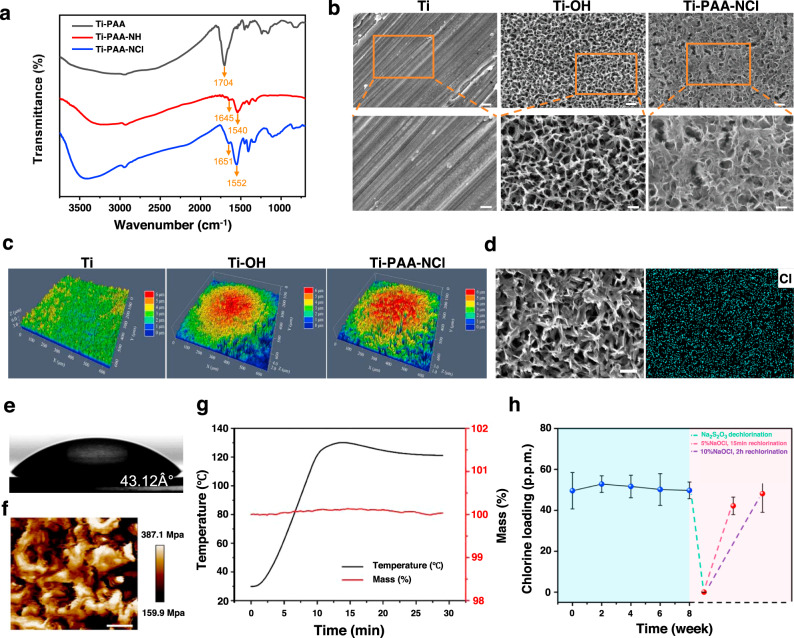
Structure characterization of the porous N-halamine polymeric coating on the titanium surface. **a** FTIR spectra of Ti-PAA, Ti-PAA-NH and Ti-PAA-NCl. **b** SEM images showing the surface morphology of Ti, Ti-OH and Ti-PAA-NCl (upper scale bars = 400 nm, lower scale bars = 200 nm). **c** CSLM images of the surface roughness of Ti, Ti-OH and Ti-PAA-NCl. **d** Elemental mapping of Ti-PAA-NCl showing the homogeneous distribution of Cl in the pore wall of N-halamine polymeric coating (scale bar = 5 μm). **e** Water contact angle of Ti-PAA-NCl. **f** Young’s modulus mapping via AFM for the N-halamine polymeric coating of Ti-PAA-NCl (scale bar = 200 nm). **g** TG curve of Ti-PAA-NCl showing good thermal stability. **h** Storage stability and regeneration of oxidative Cl^+^ for Ti-PAA-NCl (*n* = 3). The pink part gives the Cl^+^ contents of the dechlorinated and rechlorinated products of Ti-PAA-NCl with 8 weeks of storage. Error bars = s.d.

We construct the porous N-halamine polymeric coating on the titanium surface through surface pore-making, surface grafting and N-Cl functionalization (Fig. 1e). Surface pore-making via alkali-heat treatment provides the titanium surface with well-developed porosity and high surface area so as to graft as many N-halamine polymeric chains as possible during the subsequent surface grafting of polyacrylic acid (PAA). The samples obtained by surface pore-making and surface grafting are referred to as Ti-OH and Ti-PAA, respectively. For N-Cl functionalization, Ti-PAA is reacted with excess ethanediamine and sodium hypochlorite (NaOCl), which yields the aminated sample Ti-PAA-NH and the targeted product Ti-PAA-NCl with the porous N-halamine polymeric coating, respectively. The validity of N-Cl functionalization is supported by Fourier transform infrared (FTIR) spectra in Fig. 2a. The peak at 1704 cm^−1^ in Ti-PAA, representing the C = O absorption bands from the carboxyl groups of PAA^21^, disappears in Ti-PAA-NH, and two new peaks at 1645 and 1540 cm^−1^, resulting from the C = O stretching vibration and N−H bending vibration, respectively^22^, occur in Ti-PAA-NH. Furthermore, the vibration peak of C = O is shifted to 1651 cm^−1^ in Ti-PAA-NCl, because of an inductive effect of N-Cl groups^23,24^. Gel permeation chromatography shows that the N-halamine polymer chains cleaved from Ti-PAA-NCl have a weight-average molecular weight (*M*_w_) of 17,140. These results clearly reveal that N-halamine polymers are successfully grafted on the titanium surfaces.

Scanning electron microscopy (SEM) images in Fig. 2b show the surface morphology of Ti-PAA-NCl and its ungrafted precursors. Different from the nonporous surface of its untreated titanium with a low surface roughness (Psa value = 0.49, Fig. 2c and Supplementary Fig. 1a), the alkali-heat-treated Ti-OH presents a honeycomb-like porous surface morphology with pore diameters of ~200–400 nm (Fig. 2b), and has a much larger surface roughness of 0.69 (Fig. 2c and Supplementary Fig. 1a). Such a porous morphology is inherited well in Ti-PAA-NCl because of conformal coating by surface grating (Fig. 2b), which can promote osteoblast differentiation, proliferation and bone formation^25,26^. This good pore morphology retention capacity is also supported by the close surface roughness between Ti-OH and Ti-PAA-NCl (0.69 vs 0.75, Fig. 2c and Supplementary Fig. 1a). Moreover, the Ti-OH-templated N-halamine polymeric surface layer of Ti-PAA-NCl has decreased pore diameters of ~100–300 nm, since the introduction of homogeneous coating thickens the pore walls. Iodometric/thiosulfate titration measurement shows Ti-PAA-NCl has an antibacterial oxidative chlorine (Cl^+^) content of 49.57 p.p.m., and the elemental mapping in Fig. 2d demonstrates that these chlorine elements are uniformly distributed on the pore walls of Ti-PAA-NCl. In addition, other surface parameters including contact angle and Young’s modulus for the coating of Ti-PAA-NCl are measured to be 43.12° (Fig. 2e and Supplementary Fig. 1b) and 261 MPa (Fig. 2f), respectively.

### Stability and renewability of N-halamine polymeric coating

Sterilization of the implants is a vital step before insertion. Compared to irradiation (E-beam and Gamma), plasma, chemicals (peracetic acid) and many other modalities, heat sterilization has proved to be an efficient, convenient and low-cost approach for the implants^27^. So, we test the thermal stability of Ti-PAA-NCl under the temperature of common heat sterilization for medical products. The measurement temperature is increased from room temperature to 121 °C and then maintained for 20 min (ISO 17665-2:2009). The thermogravimetric (TG) curve shows that the weight percentage of Ti-PAA-NCl stays at ~100% (Fig. 2g) in the overall heat treatment process, demonstrating that the thermal stability of N-halamine polymeric coating of Ti-PAA-NCl is good enough to resist the high treatment temperatures of practical heat sterilization.

Considering the dental implants will not be used immediately after they are produced by manufactories, we need to ensure that Ti-PAA-NCl has good storage stability. So, we simulate the storage stability by testing the Cl^+^ content of Ti-PAA-NCl samples with different storage times. As the storage time increases to 8 weeks, the available Cl^+^ content of Ti-PAA-NCl remains almost unchanged (Fig. 2h, blue part), illustrating that Ti-PAA-NCl has good storage stability. After that, in order to reveal the renewability of Ti-PAA-NCl, we use excess sodium thiosulfate (Na_2_S_2_O_3_) solution to consume the oxidative Cl^+^ of Ti-PAA-NCl with 8 weeks of storage. We find that the dechlorinated product can be restored to the original level by being immersed in 10% NaOCl solution for 2 h (Fig. 2h, pink part). More importantly, for better practicability in operation, we also make rechlorination by irrigating the dechlorinated product with 5% NaOCl solution with a tailored pH of 7 for as short as 15 min, and find that its rechlorination effectiveness is as high as 88% of that under the former tough immersion condition (Fig. 2h, pink part). These results demonstrate that Ti-PAA-NCl can be regenerated in a simple manner after consumption.

### High antibacterial activity

**Fig. 3:**
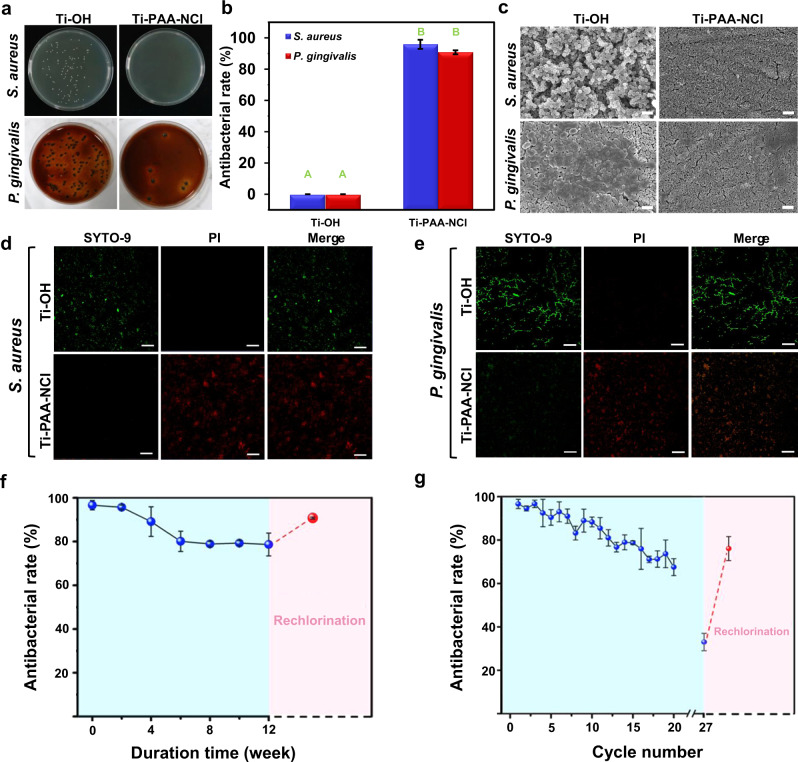
Antibacterial assessments. **a** Images of the bacterial colonies formed by *S. aureus* and *P. gingivalis* contacted with Ti-OH and Ti-PAA-NCl. **b** Antibacterial rates against *S. aureus* and *P. gingivalis* contacted with Ti-OH and Ti-PAA-NCl. Significant differences between Ti-OH and Ti-PAA-NCl are marked by different letters (*n* = 3; *P* < 0.0001; Student’s *t* test). **c** SEM images of *S. aureus* and *P. gingivalis* on the surfaces of Ti-OH and Ti-PAA-NCl (scale bar = 2 μm). Fluorescent images exhibiting the live/dead distribution of **d** *S. aureus* and **e** *P. gingivalis* on the surfaces of Ti-OH and Ti-PAA-NCl (green for live cells, red for dead cells, scale bar = 50 μm). **f** Quantitative measurements of long-lasting and renewable antibacterial rates of Ti-PAA-NCl via CFU counting after samples were stored in PBS for different duration times (*n* = 3). The pink part gives the antibacterial rate of the rechlorinated product of Ti-PAA-NCl with 12 weeks of durations. **g** Quantitative measurement of the repeated and renewable antibacterial activities of Ti-PAA-NCl via CFU counting (*n* = 3). The pink part gives the antibacterial rate of the rechlorinated product of Ti-PAA-NCl with 27 cycles. All error bars = s.d.

The colonization of bacteria is crucial to the formation of peri-implant infection. Once the bacteria produce biofilms, they will elude innate and adaptive host defences^28^, and the effect of clinical therapy will be dramatically reduced because bacteria are protected by extracellular matrix and gene mutations can be induced, causing drug resistance. Therefore, immediate action is required to avoid bacteria and biofilm accumulation^29^, meaning that antibacterial and antibiofilm capability of implants is of great importance. Herein, *Staphylococcus aureus* (*S. aureus*) and *Porphyromonas gingivalis* (*P. gingivalis*) are selected as model bacteria since they are two main pathogenic bacteria responsible for peri-implant infection, representing aerobe and anaerobe, respectively^5,30^. According to the release killing assay results, the average rate of anti-*S. aureus* in a medium is 64%, while that of anti-*P. gingivalis* is 42% (Supplementary Fig. 2a, b), indicating that before bacteria get close to the implant surface, Ti-PAA-NCl can effectively reduce bacteria invasion and prevent peri-implant infection by releasing effective antibacterial components to the surrounding environment. If the invasive bacteria break through the above defensive barrier and come into contact with the coating surface of Ti-PAA-NCl, 96% of *S. aureus* and 91% of *P. gingivalis* will be wiped out, comparable to many other antibacterial modification methods on titanium surface^9,31–33^, as shown in the contact killing assay results of Fig. 3a, b. SEM images in Fig. 3c further exhibit that the number of colonies on the surface of Ti-OH derived from *S. aureus* or *P. gingivalis* is significantly higher than that on the surface of the antibacterial Ti-PAA-NCl. Bacteria on the Ti-OH surface maintain intact cellular morphology with a smooth surface and accumulate to form biofilms. As a stark contrast, bacteria on the surface of Ti-PAA-NCl are diffusely distributed, and the appearance of the bacteria is extraordinarily distorted and incomplete. Furthermore, in order to monitor the viability of bacterial populations after contacting with Ti-PAA-NCl, we carry out bacterial fluorescent staining. We find that almost all of the *S. aureus* and *P. gingivalis* attached to the Ti-OH surface are stained fluorescent green, suggesting that they have intact cell membranes and are alive. In sharp contrast, *S. aureus* and *P. gingivalis* adhered to the Ti-PAA-NCl surface are nearly all stained fluorescent red, indicating that they are dead with damaged membranes (Fig. 3d, e). Overall, the results reveal that due to the construction of N-halamine polymeric coating, our Ti-PAA-NCl can effectively kill the key bacteria of peri-implant infection and prevent the formation of bacterial biofilm.

### Long-lasting and renewable antibacterial property

Besides powerful antibacterial function, long-lasting antibacterial property of Ti-PAA-NCl is another important issue that should be considered to guarantee the effective prevention of peri-implant infection. In general, the osseointegrated interface is weakest within 4 weeks after implantation, and would not be completely formed until 3 months after implantation^34^, so the antibacterial duration should be at least 4 weeks, ideally >3 months. Taking antibacterial effect against *P. gingivalis* as a typical example, as the duration time increases in phosphate-buffered saline (PBS), the antibacterial rate of Ti-PAA-NCl decreases from original 96 to 89% after 4 weeks and remains at 79% after 12 weeks (Fig. 3f, blue part). In short, our Ti-PAA-NCl guarantees a promising antibacterial protection during the whole formation process of osseointegrated interface.

Long-lasting antibacterial property of Ti-PAA-NCl is also reflected in the cyclic antibacterial test, which is a close way for Ti-PAA-NCl to mimic the biological process of incessantly contacting with bacteria. Taking antibacterial effect against *P. gingivalis* as an example, for each cycle, Ti-PAA-NCl is first contacted with bacteria for 24 h, ultrasonically vibrated to detach bacteria for seeding on culture plates, and disinfected with ethanol. As shown in the blue part of Fig. 3g, from the first to tenth cycle, the antibacterial rate of Ti-PAA-NCl decreases slowly from 96 to 88%, and still remains 68% at the 20th cycle. These results confirm that our Ti-PAA-NCl can be continuously antibacterial when exposed to bacteria after peri-implant infection occurs.

Renewable antibacterial ability is a coveted property of the implant coatings to guarantee the treating effectiveness once peri-implantitis occurs. By simply immersing the samples in sodium hypochlorite, the antibacterial rate of Ti-PAA-NCl that has been stored in PBS for 12 weeks can be regenerated from 78 to 91% (Fig. 3f, pink part). On the other hand, we also prolong the above cyclic test to much longer cycles (i.e. 27th cycle, Fig. 3g), the antibacterial rate goes down to 33%. However, we find that by simple rechlorination in sodium hypochlorite, the antibacterial rate of Ti-PAA-NCl returns from 33 to 76% (Fig. 3g, pink part). Note that the reason why antibacterial rate can’t be completely restored to its original level in the cyclic test may be that the mechanical force of ultrasonic vibration in each cycle destroys the grafted functional polymeric chains of Ti-PAA-NCl. That is to say, Ti-PAA-NCl could have a better renewable antibacterial property in potential dental implant applications, because of no extra mechanical force. Altogether, our Ti-PAA-NCl has a good renewable antibacterial property and thus is expected to be effective in the treatment of peri-implantitis.

### Antibacterial performance against complex bacteria from patients with peri-implantitis

**Fig. 4:**
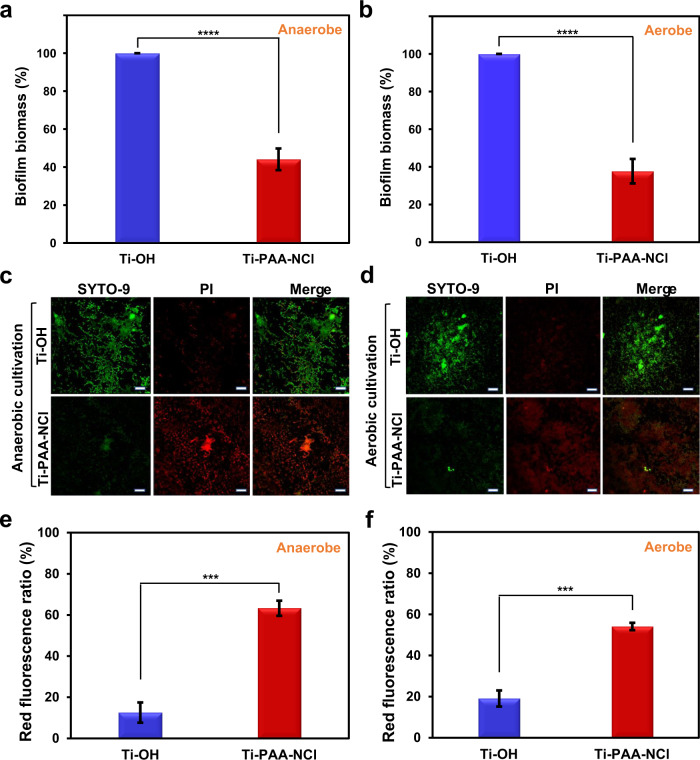
Antibacterial effect on complex bacteria from patients with peri-implantitis after 24 h incubation. The amount of biofilm biomass shown as a percentage of that formed on Ti-OH under **a** anaerobic and **b** aerobic cultivation evaluated by crystal violet staining (*n* = 10). Fluorescent staining illustrating the live/dead distribution of **c** anaerobic bacteria and **d** aerobic bacteria (green for live cells, red for dead cells; scale bars = 20 μm). Quantitative analysis of the live/dead staining results for **e** anaerobic and **f** aerobic bacteria (*n* = 3). ***P < 0.001, ****P < 0.0001; Student’s *t* test; all error bars = s.d.

The above results have proved that our Ti-PAA-NCl has a robust biocidal activity to a single kind of key pathogenic bacteria responsible for peri-implant infection, such as *S. aureus* and *P. gingivalis*. Next, we further reveal how our Ti-PAA-NCl exerts antibacterial effects on the complex bacteria of peri-implant infection. First of all, we collect microbes from patients who are clinically diagnosed with peri-implantitis, and immediately use them for the test, which will provide a more accurate reflection about the capacity of Ti-PAA-NCl to control and treat existing peri-implant infection, compared with the above single type of bacteria and even with the model system of complex oral microbiome^17^. Considering the complex bacteria of peri-implantitis include both anaerobic and aerobic bacteria^35,36^, we establish anaerobic and aerobic conditions, respectively, to assess the antibacterial effect of Ti-PAA-NCl. As shown in Fig. 4a, b, Ti-PAA-NCl reduces 56% and 62% of biofilm biomass for anaerobic and aerobic bacteria, respectively, which are comparable to other detection of antibacterial effect against complex bacteria^17,37^. Moreover, the fluorescent staining further reveals that both anaerobic and aerobic bacteria on the surface of Ti-OH are mainly stained with bright fluorescent green suggesting alive bacteria, whereas those on the surface of Ti-PAA-NCl are mostly stained with fluorescent red illustrating dead bacteria (Fig. 4c, d). The percentages of dead bacteria, described by the red fluorescence ratios, are 13% on Ti-OH and 63% on Ti-PAA-NCl for anaerobic bacteria (Fig. 4e), and 19% on Ti-OH and 54% on Ti-PAA-NCl for aerobic bacteria (Fig. 4f). The impressive anti-biofilm and antibacterial performance of our Ti-PAA-NCl against complex bacteria of peri-implantitis can greatly reduce the overall invasiveness of bacteria, which will reduce the burden on the host’s phagocytic leucocytes self-defence system and provide a great chance of successful reversal of peri-implantitis.

### Biocompatibility assessment

**Fig. 5:**
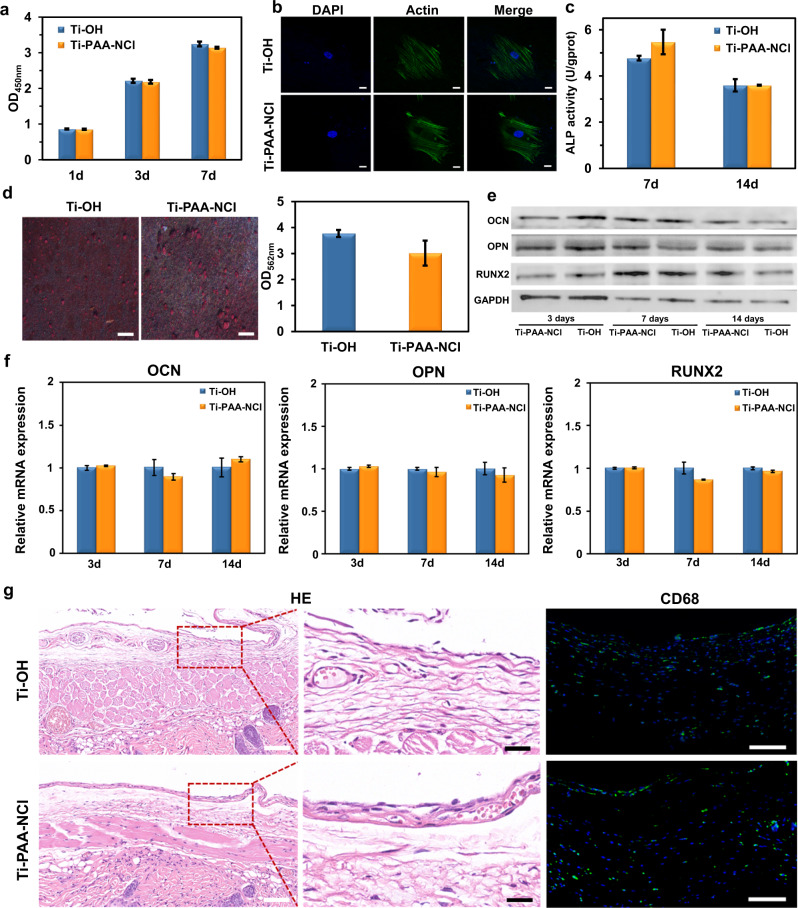
Biocompatibility assessments. **a** CCK-8 assay about the proliferation of MC3T3-E1 preosteoblasts cultured on Ti-OH and Ti-PAA-NCl after cells were cultured for 1, 3 and 7 days (*n* = 3; *P* = 0.673, 0.639 and 0.145 for 1, 3 and 7 days compared with Ti-OH, respectively; Student’s *t* test). **b** Morphology of MC3T3-E1 preosteoblasts cultured on Ti-OH and Ti-PAA-NCl for 1 day (green for F-actin, blue for cell nucleus, scale bar = 20 μm). **c** ALP activity of MC3T3-E1 preosteoblasts cultured on Ti-OH and Ti-PAA-NCl for 7 and 14 days (n = 3; *P* = 0.132 and 0.954 for 7 and 14 days compared with Ti-OH; Student’s *t* test). **d** Alizarin Red S staining of MC3T3-E1 preosteoblasts cultured on Ti-OH and Ti-PAA-NCl for 21 days (red for calcium nodules, scale bar = 1 mm) and semi-quantitative measurement of calcium content (*n* = 3; *P* = 0.147 compared with Ti-OH; Student’s *t* test). **e** Western blot results of osteogenic-related proteins (OCN, OPN and RUNX2) expressed in MC3T3-E1 preosteoblasts cultured on Ti-OH and Ti-PAA-NCl for 3, 7 and 14 days after osteogenic induction. **f** RT-qPCR results of expression levels of osteogenic-related genes (OCN, OPN and RUNX2) in MC3T3-E1 preosteoblasts cultured on Ti-OH and Ti-PAA-NCl for 3, 7 and 14 days after osteogenic induction (*n* = 3; *P* > 0.05 for all time points of these three genes between groups; Student’s *t* test). **g** In vivo biocompatibility. HE staining and CD68 immunofluorescent staining of tissues around Ti-OH and Ti-PAA-NCl embedded in the backs of nude mice for 4 weeks (fluorescent green for CD68, fluorescent blue for cell nucleus, white scale bars in HE images = 100 μm, black scale bars in HE images = 25 μm, scale bars in CD68 immunofluorescent images = 100 μm). All error bars = s.d.

Since biocompatibility is a basic requirement for biomedical materials, we carry out Cell Counting Kit-8 (CCK-8) assay to determine the cytotoxicity of Ti-PAA-NCl on cell viability and proliferation. We find that there is no significant difference in growth and proliferation of MC3T3-E1 preosteoblasts between Ti-PAA-NCl and Ti-OH groups (Fig. 5a). The fluorescent staining results further exhibit that MC3T3-E1 preosteoblasts on both Ti-OH and Ti-PAA-NCl surfaces are polygonal in shape, which are fully spread with some filopodia, and their cytoskeletons appear filamentous arranging in the same direction (Fig. 5b). Thus, these data indicate that Ti-PAA-NCl has no adverse effects on cell proliferation and adhesion.

As dental implants need to be implanted into the bone, not only the cytotoxicity but also the effect on osteogenic function is of great importance. Figure 5c shows no significant differences in the activities of alkaline phosphatase (ALP, a marker of early osteogenesis) for MC3T3-E1 preosteoblasts between Ti-OH and Ti-PAA-NCl after osteogenic induction for 7 and 14 days. In addition, Alizarin Red S staining of MC3T3-E1 preosteoblasts following osteogenic induction for 21 days exhibits that the distribution of red calcium nodules on the surface of Ti-PAA-NCl is similar to that on the surface of Ti-OH, and the semi-quantitative measurement of calcium content between two groups has no significant difference (Fig. 5d). In addition, there are still no significant differences in expression levels of osteogenic proteins and genes, such as osteocalcin (OCN), osteopontin (OPN), and Runt-related transcription factor 2 (RUNX2) for MC3T3-E1 preosteoblasts between Ti-OH and Ti-PAA-NCl (Fig. 5e, f). These results indicate that the introduction of our N-halamine polymeric coating does not change the osteogenic properties of titanium implants.

To further illustrate the biocompatibility in vivo, both Ti-OH and Ti-PAA-NCl samples are implanted into the back of nude mice for 4 weeks. All wounds are well healed, free of purulent secretion or other apparent inflammation. After dissecting the subcutaneous tissue, we find that all the samples are covered with a fibrotic capsule. The infiltration of inflammatory cells in the fibrotic capsule is assessed by haematoxylin and eosin (HE) staining and the distribution and number of macrophages are evaluated via CD68 immunofluorescent staining (Fig. 5g). HE staining clearly shows that there is no significant difference in both the thickness of fibrotic capsule and the infiltration extent of inflammatory cells between Ti-OH and Ti-PAA-NCl groups. Moreover, CD68-positive macrophages distribute sporadically without obvious aggregation or quantitative variances in both Ti-OH and Ti-PAA-NCl groups. Taken together, these results indicate that our Ti-PAA-NCl has good in vivo biocompatibility and does not cause significant inflammation or rejection.

### In vivo osseointegration and anti-infection ability

**Fig. 6:**
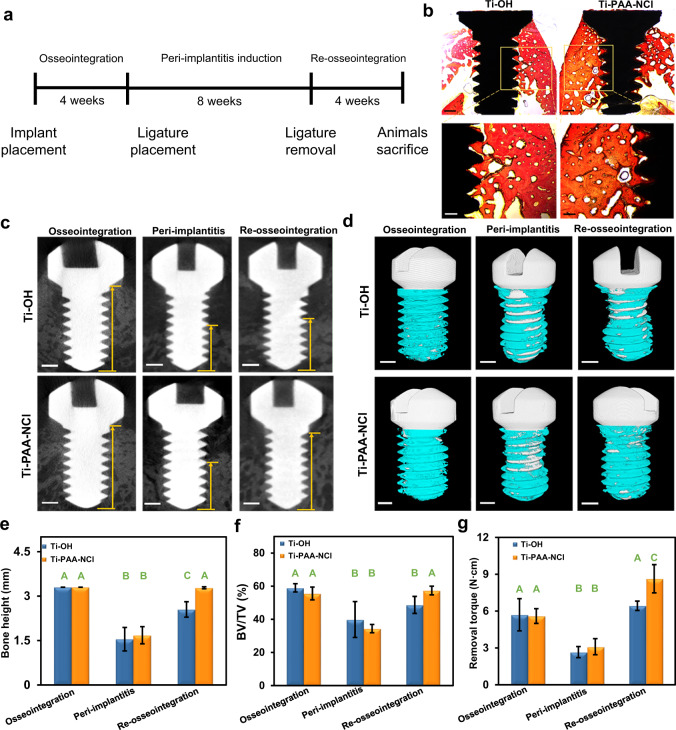
In vivo assessments of osseointegration ability and effect against peri-implant infection. **a** Schematic timetable of experimental design. **b** Longitudinal sections of non-decalcified specimens stained with Van Gieson’s staining after 4 weeks of implantation (upper scale bars = 500 µm, lower scale bars = 250 µm). **c** Micro-CT 2D image analysis illustrating the bone height surrounding the implants after osseointegration for 4 weeks, peri-implantitis for 8 weeks and re-osseointegration for 4 weeks (the length of yellow arrows represents the bone height referring to the vertical distance between the implant tip and the marginal bone level below the implant shoulder, scale bars = 500 µm). **d** Micro-CT 3D reconstructions of the implants and surrounding bone tissues (white for the implant, cyan for bone tissues, scale bars = 500 µm). **e** Height and **f** BV/TV of the bone surrounding implants, and **g** maximum removal torque of implants at all time points (*n* = 3; different letters mean *P* < 0.05 for Ti-OH or Ti-PAA-NCl group among different time points and within each time point between groups; Wilcoxon nonparametric test or Student’s *t* test; error bars = s.d.).

In order to assess in vivo osseointegration ability of our coating, titanium mini-implants with Ti-OH and Ti-PAA-NCl surfaces are implanted into bilateral edentulous areas of mandibles in New Zealand white rabbits (Supplementary Fig. 3a–f). We evaluated the newly formed bone surrounding implants via Van Gieson’s staining after implantation for 4 weeks (Fig. 6a). The result shows that mini-implants both with Ti-OH and Ti-PAA-NCl surfaces can form satisfactory osseointegration (Fig. 6b), suggesting that surface modification with our coating does not affect the osseointegration viability and bone compatibility of titanium implants.

Furthermore, to explore the long-lasting anti-infection ability of our coating, the rabbit model of ligature-induced peri-implantitis is built after 4 weeks of osseointegration (Supplementary Fig. 3g, h). Ligatures are removed after 8 weeks of ligation, and then implants are allowed to progress undisturbedly for 4 weeks (Fig. 6a). 2D analysis and 3D reconstruction of the micro-computed tomography (micro-CT) data show that both the bone height and the ratio of bone volume to total volume (BV/TV) surrounding implants with 8 weeks of ligation decrease significantly, meaning the successful induction of peri-implantitis (Fig. 6c–f). After the ligatures are removed for re-osseointegration for 4 weeks, the bone height and BV/TV surrounding Ti-PAA-NCl implants have almost risen to the original level of osseointegration, while those surrounding Ti-OH implants remain at a relatively low level, although with slight improvement (Fig. 6c–f). In addition to radiographic analysis, biomechanical evaluation is also conducted to quantitatively detect the bonding force between implant and bone. As shown in Fig. 6g, when ligatures exist, peri-implantitis constantly occurs, sharply weakening the bonding force between bone and implant. After ligatures are removed, the bonding forces become strong in both Ti-OH and Ti-PAA-NCl, but Ti-PAA-NCl exhibits a greater enhancing effect, which displays the same tendency as the radiographic results. All these results prove that Ti-PAA-NCl can achieve a prolonged anti-infection effect and promote the recovery of bone tissue that is previously resorbed in peri-implantitis.

### Long-lasting and renewable antibacterial and anti-biofilm performance against human intraoral bacterial colonies

**Fig. 7:**
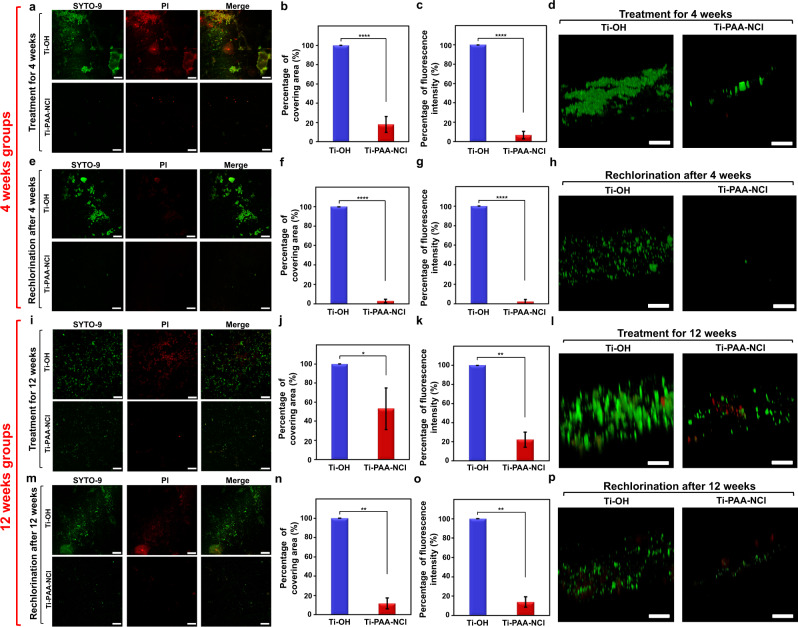
Intraoral assessment of the long-lasting and renewable antibacterial effect against human bacterial colonies. **a** Fluorescent staining of bacteria on Ti-OH and Ti-PAA-NCl after treatment in the complex environment of the real oral cavity for 4 weeks. **b**, **c** Percentages of **b** covering area and **c** fluorescence intensity of bacteria on Ti-OH and Ti-PAA-NCl after treatment for 4 weeks. **d** 3D morphology of the fluorescently stained biofilms on Ti-PAA-NCl and Ti-OH after treatment for 4 weeks. **e**–**p** Fluorescent staining, quantitative analysis and biofilm morphology of bacteria on Ti-PAA-NCl and Ti-OH **e**–**h** after treatment for 4 weeks and rechlorination, **i**–**l** after treatment for 12 weeks and **m**–**p** after treatment for 12 weeks and rechlorination. Green for live bacteria and red for dead bacteria; scale bars in **a**, **e**, **i**, **m** = 50 μm, scale bars in **d**, **h**, **l**, **p** = 20 μm; *n* in **a**–**h** = 8, *n* in **i**–**p** = 3. *P < 0.05, **P < 0.001 and ****P < 0.0001; Student’s *t* test; all error bars = s.d.

Based on the good biocompatibility, Ti-PAA-NCl is further placed in the real environment of the human oral cavity to veritably detect the long-lasting and renewable antibacterial properties against human intraoral bacterial colonies. Innovatively, tiny titanium disks with Ti-OH and Ti-PAA-NCl surfaces are bonded on the buccal surfaces of upper and lower first molars in volunteers’ mouths, allowing the coatings to really experience the daily challenges such as eating, tooth brushing and so on (Supplementary Fig. 4a, b). The result shows that after 4 weeks placement in intraoral environment, the covering area and fluorescence intensity of bacteria on Ti-PAA-NCl are only 18% and 7% of those on Ti-OH, respectively (Fig. 7a–c). Therefore, not surprisingly, biofilms are discrete and thin on Ti-PAA-NCl, but dense and thick on Ti-OH (Fig. 7d). In order to detect the renewable antibacterial properties of Ti-PAA-NCl, we irrigated the coatings with 5% sodium hypochlorite for 15 min under the protection of a rubber dam (Supplementary Fig. 4c, d). As expected, after rechlorination, the covering area and fluorescence intensity of bacteria on Ti-PAA-NCl are as low as 3% and 2% of those on Ti-OH, respectively. As a result, there are just trace amounts of biofilms on Ti-PAA-NCl as compared to Ti-OH (Fig. 7e–h).

In order to more thoroughly demonstrate the long-lasting and renewable antibacterial properties of our coating, we extend the time of the coating kept in the mouth from 4 to 12 weeks. Satisfyingly, after exertion for 12 weeks, the covering area and fluorescence intensity of bacteria on Ti-PAA-NCl are 53% and 22% of those on Ti-OH (Fig. 7i–k), respectively, and biofilms on Ti-PAA-NCl are much sparser than on Ti-OH (Fig. 7l). In sharp contrast, after rechlorination, the covering area and fluorescence intensity of bacteria on Ti-PAA-NCl are as low as 12% and 14%, respectively (Fig. 7m–o). As such, biofilms are barely visible on Ti-PAA-NCl after rechlorination, while biofilms apparently exist on Ti-OH (Fig. 7p). The above results together demonstrate that even in the complex environment of the real oral cavity, Ti-PAA-NCl still exhibits favourable long-lasting and renewable antibacterial properties against human bacterial colonies.

## Discussion

Nowadays, many bactericidal agents have been used in antibacterial modification of biomedical materials, such as metal ions, antibiotics, antimicrobial peptides, polymeric quaternary ammonium salts and N-halamines^10–12,38^. Among them, N-halamines have attracted great interest because of their powerful antibacterial activity toward a broad spectrum of bacteria, long-term stability in both aqueous and dry conditions, renewability, good biosafety, and low costs. In the recent decade, N-halamines have been widely utilized in fields including water disinfection^39^, wound dressing^40,41^, food packaging^42^ and medical instruments^43^, but so far, they have been rarely exploited for dental implants. Therefore, our work could be an advanced example of utilization of N-halamines for antibacterial surface modification of dental implants.

N-halamines are a class of compounds bearing one or more nitrogen–halogen (N–X) bonds. The halogens in N-halamines can be chlorine, bromine or iodine, among which chlorine is the most commonly used because of the favourable stability of N–Cl bond. According to the structures, organic N-halamines can be divided into three categories: imide N-halamines [–C(O)-NX-C(O)–], amide N-halamines [–C(O)-NX-R] and amine N-halamines (RR′-NX)^24^. The dissociation constants of chlorine from N-halamine structures in aqueous solutions decrease in the following order: imide > amide > amine N-halamine^44^, which means imide N-halamine is the least stable structure that can rapidly release active Cl^+^ into the medium to kill bacteria, whereas amine N-halamine is the most stable structure with highest durability but slowest antibacterial rate. Actually, amide N-halamines are regarded as the most practical for not only industrial applications^45^ but also biomedical materials^40^, since they have a moderate transfer rate of oxidative Cl^+^ from N-halamine to bacteria in medium and thus provide reasonably rapid antibacterial effect^44^. As such, we firstly consider amide N-halamine as the primary target structure in the coating design. However, the stability of N-halamine in the coating is crucial because the long-term persistence of N-halamine is essential for preventing bacterial invasion, especially before the complete formation of osseointegrated interface. For this reason, amine N-halamine is another indispensable structure in the coating. Based on the above analysis, we utilize excess ethanediamine to react with Ti-PAA to ensure that the resulting coating contains not only amide N-halamine but also amine N-halamine after chlorination. Such a molecular design can provide a synergistic effect of both rapid and long-lasting antibacterial functions. Indeed, such a synergistic antibacterial effect is thoroughly exemplified not only in our high antibacterial and long-lasting antibacterial experiments in vitro but also in peri-implantitis animal model and human oral environment. Our N-halamine polymeric coating ensures that the contact antibacterial rate can be as high as 96%, and the antibacterial rate remains at 79% after 12 weeks. More importantly, our coating performs stable and prolonged anti-infection function in peri-implantitis animal model, as well as exhibits satisfactorily long-lasting antibacterial effect in the complex environment of the human oral cavity. These guarantee great protection in the whole formation process of osseointegrated interface, which can prevent peri-implant infection effectively at the early stage after implantation.

With the increasing application of N-halamines, many studies have focused on the mechanisms of their antibacterial actions. Normally, the antibacterial mechanism of N-halamines can be classified as contact killing and release killing, although there are few reports about transfer killing^46^. In contact killing, active halogens directly transfer from N-halamines to bacterial receptors without dissociation of halogen from N–X bonds, while the active halogens dissociate from N–X bonds firstly and then migrate to solution in release killing or transfer to the medium constituents in transfer killing. In general, N-halamines containing stable N–X bonds tend to kill bacteria via the mechanism of contact killing, and those with less stable N–X bonds act in the mechanism of release killing. In our work, the results show that the antibacterial action of our N-halamine polymeric coating occurs through combined mechanism of contact and release killing, which gives the implants double antibacterial protection. Moreover, as the structures of both chlorinated amide N-halamine and chlorinated amine N-halamine are relatively stable, the contact killing ability of our N-halamine polymeric coating is much stronger than the release killing ability. Among them, amide N-halamine structure in the coating may play a major role in the process of release killing, while the amine N-halamine structure chiefly goes with the contact killing.

Whether it is contact killing or release killing, the main component of bactericidal action for N-halamines is active halogen. Taking N-chloramine as an example, it is found that the initial attack of active Cl^+^ is to chlorinate the bacterial external protein matrix. Active Cl^+^ forms a cover around the bacterium, helping it penetrate into the bacterial cells, further oxidizes many of the key cellular constituents containing thiols and thioethers and finally denatures proteins via transchlorination^46–48^. As a result, bacteria lose their functions and die. Since N-halamines nonspecifically interact with the vital proteins of bacteria, they are considered to be sensitive toward a broad spectrum of bacteria. This is why our N-halamine polymeric coating has a good antibacterial effect on the main pathogenic bacteria of peri-implantitis, such as *S. aureus* and *P. gingivalis*, on complex bacteria from patients with peri-implantitis, and even on bacterial colonies in the real environment of the human oral cavity. In addition, from this perspective, it is not hard to imagine that even if N-halamine polymeric coating persists on the surface of the implant for a long time, it will be unlikely to induce the production of resistant bacteria.

In the above bactericidal process, the active halogen of N-halamine polymeric coating will be gradually consumed; however, they can be easily recharged and regenerate good antibacterial performance^39,42,43^. In this respect, our results have shown that active Cl^+^ can be effectively regenerated by simple rechlorination, no matter after long storage-simulating implantation and repeated antibacterial consumption in vitro, or after sustained treatment in the real oral environment. Based on the above satisfactory features of our N-halamine polymeric coating, we innovatively propose the concept of renewable antibacterial coating in dental implant. It means once peri-implantitis occurs, the antibacterial property of the implant coating can be simply and quickly regenerated to treat peri-implantitis effectively, preserving the existed implant to avoid removal of inflammatory peri-implant tissues and loss of the dental implant. This will be an innovative perspective on both antibacterial modification of implant surface and treatment of peri-implantitis.

In conclusion, we have successfully developed porous N-halamine polymeric coating on the titanium surface via surface pore-making and surface grafting, followed by N-Cl functionalization. The as-obtained Ti-PAA-NCl products have good antibacterial effects not only on the main pathogenic bacteria but also complex bacteria of peri-implant infection. More importantly, based on the well-orchestrated structural design, the antibacterial effect of our Ti-PAA-NCl can persist throughout the whole process of forming the osseointegrated interface and be easily renewed after consumption. Due to the perfect combination of long-lasting and renewable antibacterial properties, our Ti-PAA-NCl shows a very appealing application prospect for both prevention and treatment of peri-implant infection, which will ultimately contribute to the significantly enhanced success rate of dental implants.

## Methods

### Preparation of porous N-halamine polymeric coating on titanium surfaces

Titanium disks with 9.5 mm in diameter and 0.3 mm in thickness were polished stepwise with different SiC sandpapers from 400# to 1500#, ultrasonically cleaned with acetone, ethanol and distilled water each for 15 min, and then air-dried. The alkali-heat treatment was performed with 5 M NaOH for 24 h at 60 °C, and disks were immersed in distilled water and dried under vacuum.

Alkali-heated titanium disks (Ti-OH) were soaked in a silane-coupling agent of KH570 at 40% concentration for 30 min at room temperature to introduce C = C bonds onto the surface. Then, samples were transferred to a reaction bottle with ethanol, acrylic acid and azobisisobutyronitrile, and heated to 60 °C in an oil bath for 24 h under the protection of nitrogen to graft PAA. Subsequently, samples were sequentially cleaned with 1,4-dioxane and ethanol and then dried in air. The resulting Ti-PAA products were immersed overnight in a large excess of ethanediamine^49,50^ at 80 °C to undergo the reaction with 2-chloro-4,6-dimethoxy-1,3,5-triazine for 24 h. Then, samples were washed three times with ethanol and air-dried, yielding Ti-PAA-NH products. To convert –NH– and –NH_2_ groups into N-halamines, the Ti-PAA-NH samples were immersed in a large excess of NaOCl solution containing 10% active chlorine in an ice bath for 2 h under constant shaking^51^, washed with ethanol and distilled water, and then air-dried, which yielded the targeted Ti-PAA-NCl products. Note that utilization of a large excess of NaOCl guaranteed that sufficient N–H bonds could be converted to N–Cl bonds, although there could still be many residual –NH– and –NH_2_ groups in the Ti-PAA-NCl products.

### Structure characterization

FTIR spectra were recorded on FTIR spectrometer (Nicolet 6700, Thermo Scientific, USA) to analyse chemical groups of samples. SEM (S-4800, Hitachi, Japan) was used to analyse the surface morphology of samples. Roughness measurements were performed by the confocal laser scanning microscope (CLSM; LSM 700, Zeiss, Germany) and the parameter of Psa was measured as the average of the 3D roughness. Elemental mapping was performed using SEM (ΣIGMA 300, Zeiss, Germany) with an energy dispersive spectrometer (AZtecLive, Oxford Instrument, UK) to detect the element distribution of the N-halamine polymeric coating. Water contact angle measurements were conducted using a drop shape analyser (DSA 100, Kruss GmbH, Germany) by dropping with pure water to evaluate the hydrophilicity of the different titanium surfaces. Young’s modulus of the N-halamine polymeric coating was measured by atomic force microscopy (Dimension FastScan, Bruker, Germany) in the peak force quantitative nanomechanics mode and was analysed by the Derjaguin–Muller–Toporov model.

### Stability and renewability assessments

Using a synchronous thermal analyser (STA449F5, Netzsch, Germany), thermal gravimetric analysis (TGA)^52^ was performed to test the thermal stability of samples. For storage stability, Ti-PAA-NCl samples were stored in the dark for different times (0, 2, 4, 6, and 8 weeks), and the oxidative Cl^+^ content was determined with a standard iodometric/thiosulfate titration procedure^40^. After being stored for 8 weeks, samples were completely quenched with excess Na_2_S_2_O_3_ solution to consume the Cl^+^ and then treated with NaOCl again to detect the renewable ability of Ti-PAA-NCl. The weight percent of Cl^+^ on Ti-PAA-NCl samples was determined from the equation below:

1 $$\text{Cl}^{+}\;(\text{p.p.m.})=\frac{{C}\times \text{V}\times 35.45}{{W}\times 2}\times {10}^{6}$$

where *C* and *V* represent the molar concentration (mol L^−1^) and volume (L) of the Na_2_S_2_O_3_ consumed in the titration, respectively, and *W* represents the weight (g) of each sample.

### Antibacterial activity assessments

The antibacterial effect of Ti-PAA-NCl was evaluated with aerobe (*S. aureus*, ATCC25923, Guangdong Microbial Culture Collection Centre, China) and anaerobe (*P. gingivalis*, ATCC33277, Guangdong Microbial Culture Collection Centre, China). *Staphylococcus aureus* was cultured overnight at 37 °C in Luria Bertani (LB) growth medium (10 g of tryptone L^−1^ and 5 g of yeast extract L^−1^) in a shaking incubator. *Porphyromonas gingivalis* was cultured in brain heart infusion (BHI) broth at 37 °C in anaerobic environment (80% N_2_, 10% H_2_ and 10% CO_2_). The antibacterial abilities were assessed by the plate counting method, in which the number of colony-forming units (CFUs) on agar plates was determined^53^. In brief, samples were placed in 48-well plate, immersed in 300 μL of *S. aureus* or *P. gingivalis* suspension (10^7^ CFU mL^−1^) and incubated at 37 °C for 12 h. First, to determine the release killing ability of the coating, the above culture medium containing *S. aureus* or *P. gingivalis* was diluted 10,000-fold with PBS. Twenty microlitres of the dilute bacterial suspension was seeded on agar plates for *S. aureus* and on BHI agar plates containing 10% sheep blood for *P. gingivalis*. Next, to detect the contact killing ability of Ti-PAA-NCl, bacteria adhered to the surfaces of samples were detached by ultrasonication (300 W, 40 kHz) in 1 mL PBS for 3 min. Bacteria suspension was diluted 10,000-fold for *S. aureus* and 1000-fold for *P. gingivalis* with PBS, and then seeded on plates as above. Both release- and contact-type antibacterial rates were calculated by the following formula:

2 $${\rm{Antibacterial}}\;\;{\mathrm{rate}}\;( \% )=\frac{{\text{CFU}} \,{\text{of}}\, {\text{Ti}}{\hbox{-}}{\text{OH}}-{\text{CFU}} \,{\text{of}}\, {\text{Ti}}{\hbox{-}}{\text{PAA}}{\hbox{-}}\text{NCl}}{{\text{CFU}} \,{\text{of}}\, {\text{Ti}}{\hbox{-}}\text{OH}}\times 100$$

For SEM imaging, 500 μL of *S. aureus* or *P. gingivalis* suspension (10^7^ CFU mL^−1^) was seeded onto different samples and cultured for 12 h at 37 °C. Then, samples were rinsed with PBS, immersed in 2.5% glutaraldehyde overnight at 4 °C, and sequentially dehydrated with gradient ethanol. Finally, samples were coated with gold and analysed with SEM (S-4800, Hitachi, Japan). For fluorescent imaging, the viability of bacteria was detected by LIVE/DEAD *Bac*Light Bacterial Viability Kit (Molecular Probes Inc., Eugene, USA). After seeding the bacteria for 12 h, samples were washed with PBS, stained for 15 min in the dark with LIVE/DEAD *Bac*Light Bacterial Viability Kit and visualized with CLSM (LSM 780, Zeiss, Germany).

### Long-lasting and renewable antibacterial effect assessments

The long-lasting antibacterial abilities were evaluated using *P. gingivalis* through two methods. First, Ti-OH and Ti-PAA-NCl samples were stored in PBS for different durations (0, 2, 4, 6, 8, 10 and 12 weeks). They were then transferred to 24-well plate with 400 μL of 10^6^ CFU mL^−1^ *P. gingivalis* suspension in each well and incubated at 37 °C under anaerobic conditions for 24 h. Bacteria adhered to the surfaces of samples in 1 mL PBS were detached by ultrasonication (300 W, 40 kHz) for 3 min. Bacteria suspension was diluted 1000-fold with PBS and seeded on plates. Second, the cyclic antibacterial effect of Ti-OH and Ti-PAA-NCl samples was tested. Briefly, in each cycle, the samples were immersed in 400 μL of 10^6^ CFU mL^−1^ *P. gingivalis* suspension for 24 h and then bacteria adhered to the surfaces of samples were seeded on plates after ultrasonication as mentioned above. After disinfecting with 75% ethanol for 24 h, samples were resubjected to antibacterial test until the 27th cycle.

After storing in PBS for 12 weeks or the 27th cycle of antibacterial test, samples were regenerated in 10% NaOCl solution in an ice bath for 2 h and the new antibacterial rates were determined. The antibacterial rates were calculated by the following formula:

3 $${\text{Antibacterial}}\;\;{\text{rate}}\;( \% )=\frac{{\text{CFU}}\, {\text{of}}\, {\text{Ti}}{\hbox{-}}{\text{OH}}-{\text{CFU}}\, {\text{of}}\, {\text{Ti}}{\hbox{-}}{\text{PAA}}{\hbox{-}}{\text{NCl}}}{{\text{CFU}}\, {\text{of}}\, {\text{Ti}}{\hbox{-}}\text{OH}}\times 100$$

### Assessments of antibacterial performance against complex bacteria from patients with peri-implantitis

Bacteria were collected from peri-implant pockets of patients with peri-implantitis, and the procedures were performed under the permission of the Ethics Committee of Hospital of Stomatology, Sun Yat-sen University (No. KQEC-2019-42). Inclusion criteria for peri-implantitis patients were shown in Supplementary Table 1. We used a small sterile brush to access the peri-implant pocket and collected subgingival plaque, which was immediately transferred to LB growth medium and BHI broth, respectively. Plaque suspensions were diluted to 10^6^ CFU mL^−1^ by measuring OD at 600 nm. Next, Ti-OH and Ti-PAA-NCl samples were placed in 24-well plates, and 400 μL of plaque suspension was added to each well. Samples were incubated at 37 °C for 24 h under aerobic and anaerobic conditions, respectively, stained by 0.5% crystal violet for 20 min, and then rinsed with PBS to ensure complete removal of residual dye. Three hundred microlitres of 95% alcohol was added to elute the bound crystal violet. Afterwards, the OD value of eluates was determined on the microplate reader (Epoch 2, BioTek, USA) at 595 nm, and then biofilm biomass (%) was calculated according to the following formula:

4 $${\text{Biofilm}}\;\;{\text{biomass}}\;( \% )=\frac{{\text{OD}}_{595}\;{\text{of}}\; {\text{Ti}}{\mbox{-}}\text{PAA}{\hbox{-}}\text{NCl}}{{\text{OD}}_{595}\;{\text{of}}\; {\text{Ti}}{\hbox{-}}\text{OH}}\times 100$$

Besides, bacteria on samples were stained with LIVE/DEAD *Bac*Light Bacterial Viability Kit and examined by CLSM as before. The red fluorescence ratio was quantitively analysed using the Image J software (v1.6.0, National Institute of Health, Bethesda, USA) according to the following formula:

5 $${\rm{Red}}\;{\rm{fluorescence}}\;{\rm{ratio}}\;( \% )=\frac{{\rm{Intensity}}\,{\rm{of}}\,{\rm{red}}\,{\rm{fluorescence}}}{{\rm{Intensity}}\,{\rm{of}}\,{\rm{red}}\,{\rm{and}}\,{\rm{green}}\,{\rm{fluorescence}}}\times 100$$

### Biocompatibility assessments

The proliferations of MC3T3-E1 preosteoblasts (Chinese Academy of Sciences) on Ti-OH and Ti-PAA-NCl samples were measured at the 1st, 3rd and 7th days with CCK-8 (Dojindo, Tokyo, Japan) and OD was determined on a microplate reader (Epoch 2, BioTek, USA) at 450 nm. Fluorescent staining was carried out to detect cell adhesion on coatings. Briefly, after cultured with MC3T3-E1 preosteoblasts for 1 day, samples were fixed with 4% paraformaldehyde and rinsed with PBS. After permeabilized with 1% Triton X-100, samples were stained with 4′,6-diamidino-2-phenylindole (DAPI; Beyotime, Shanghai, China) and Actin-Tracker Green (Beyotime, Shanghai, China), and finally visualized with CLSM (LSM780, Zeiss, Germany).

The osteogenic abilities of the MC3T3-E1 preosteoblasts on coatings were evaluated by ALP activity and calcium content as well as expression levels of osteogenic proteins and genes. The activity of ALP was measured using ALP Assay Kit (Jiancheng, Nanjing, China) on the 7th and 14th day of osteogenic induction. At each time point, samples were rinsed with PBS and lysed with 1% Triton X-100. Then, the lysates were transferred to 96-well plate and ALP activity was determined. The formation of calcium phosphate was detected using Alizarin Red S staining (Cyagen, Suzhou, China). After osteogenic induction for 21 days, cells on samples were fixed in 4% paraformaldehyde, rinsed with PBS, stained with Alizarin Red solution for 15 min, washed with deionized water and finally observed by stereomicroscope (MZ10F, Leica, Germany). Semi-quantitative measurement of the calcium content was then determined on a microplate reader (Epoch 2, BioTek, US) at 562 nm. The expression levels of osteogenic-related genes including OCN, OPN and RUNX2 were detected through quantitative reverse transcription-PCR (RT-qPCR) at the 3rd, 7th and 14th day after osteogenic induction. Total RNA was extracted from MC3T3-E1 preosteoblasts on samples at each time point, 500 ng of which was used to synthesize complementary DNAs (cDNAs) by PrimeScript RT Reagent Kit (Takara, Dalian, China) for reverse transcription. Then, 10 µL RT-qPCR reaction system with three replicates was performed using SYBR Green Master Mix (Takara, Dalian, China) on Real-Time PCR System (LightCycler 96, Roche, Switzerland) under the following conditions: cDNAs were denatured for 5 min at 95 °C, followed by 40 cycles composed of 10 s at 95 °C, 20 s at 55 °C and 20 s at 72 °C. The primer sequences were displayed in Supplementary Table 2 and glyceraldehyde 3-phosphate dehydrogenase (*GAPDH*) was set as the reference gene. At the same time, the osteogenic protein expressions of OCN, OPN and RUNX2 in MC3T3-E1 preosteoblasts were analysed by western blot at the 3rd, 7th and 14th days after osteogenic induction. Cells were suspended in radioimmunoprecipitation assay lysis buffer (Thermo Fisher Scientific, Rockford, USA) with 1% Complete Mini Protease Inhibitor Cocktail (Sigma-Aldrich, St. Louis, USA) for 30 min. After centrifugation, protein contents were detected by the Bicinchoninic Acid Protein Assay Kit (Cwbiotech, Beijing, China), and then protein samples were denatured at 99.9 °C for 10 min. Subsequently, proteins were separated by sodium dodecyl sulfate-polyacrylamide gel electrophoresis (Solarbio, Beijing, China) with 10% separation gel and 5% concentration gel. After that, proteins were blotted onto immobilon-P polyvinylidene fluoride membrane (Merck Millipore, Darmstadt, Germany) and blocked with 5% skim milk (Difco, BD, USA) for 1 h. Western blot was performed using rabbit anti-mouse OCN (Affinity Biosciences, Cincinnati, USA), OPN (Proteintech, Chicago, USA), RUNX2 (Abcam, Cambridge, UK) polyclonal antibodies and mouse-derived GAPDH monoclonal antibody (Emarbio, Beijing, China) overnight at 4 °C. After thorough washing, membranes were incubated with secondary horseradish peroxidase-conjugated goat anti-rabbit and goat anti-mouse IgG (Emarbio, Beijing, China) for 1 h at room temperature and developed through an Enhanced Chemiluminescence Imaging System (GeneGnome XRQ, Syngene, UK).

Six 5-week-old male BALB/c nude mice (20–2 g) obtained from the Laboratory Animal Center of Sun Yat-sen University (Guangzhou, China) were used for evaluating the biocompatibility of Ti-PAA-NCl in vivo under the permission of Animal Ethics Committee (AEC) of Sun Yat-sen University (No. SYSU-IACUC-2020-000204). One percent pentobarbital (Macklin, Shanghai, China) was intraperitoneally injected into nude mice (40–50 mg kg^−1^). A 1-cm incision was created on each side of the back. The skin was fully separated from the subcutaneous layer to the muscle tissue. For each nude mouse, one Ti-OH and one Ti-PAA-NCl were placed into the surface of muscle tissue on each side, and wounds were closed. After 4 weeks, animals were euthanized, and the tissues surrounding samples were removed and fixed in 4% paraformaldehyde. After completely dehydrated and embedded in paraffin, tissues were cut to 4 μm sections, deparaffinized in sequential xylene baths and rehydrated in graded alcohol baths. Sections were stained with HE (Beyotime, Shanghai, China) and observed with a light microscope (Axioplan, Zeiss, Germany). For immunofluorescent staining, after deparaffinization and rehydration, sections were blocked with 10% donkey serum for 20 min at room temperature and incubated with rabbit anti-mouse CD68 polyclonal antibody (Abcam, Cambridge, England) overnight at 4 °C. Sections were then incubated in Alexa Fluor 488-conjugated donkey anti-rabbit IgG (H+L) (Invitrogen, Carlsbad, USA) for 30 min at 37 °C and stained with DAPI for 10 min at room temperature, followed by observation in a fluorescent scanner (Pannoramic MIDI, 3DHistech, Hungary).

### Assessments of in vivo osseointegration and anti-infection abilities

Three-month-old male New Zealand white rabbits (2.0–2.5 kg) were used for in vivo experiment under the permission of AEC of Sun Yat-sen University (No. SYSU-IACUC-2020-000279). According to the surface modification procedures of titanium disks, Ti-OH and Ti-PAA-NCl surfaces were introduced into custom-made titanium mini-implants with thread portions of 1.6 mm in diameter and 3.3 mm in length. For each rabbit, after intravenously anaesthetized with 20% urethane (1 g kg^−1^), two mini-implants with Ti-OH surfaces were implanted into the edentulous area of the left mandible, while two mini-implants with Ti-PAA-NCl surfaces into the edentulous area of the right mandible. Then, the wounds were sutured and oral cleaning was done every other day.

To analyse the osseointegration effect, the rabbits were sacrificed after implantation for 4 weeks, and the mandibles were retrieved. Specimens were fixed in 4% paraformaldehyde for 48 h and inset in resin. Tissue blocks containing implant and surrounding soft and hard tissues were dissected to 300 µm in thickness using diamond saw microtome (SP 1600, Leica, Germany), and then grounded to 50 µm using Micro Grinding System (400 CS, EXAKT, Germany). Sections were stained with Van Gieson’s picrofuchsin and observed via stereomicroscope (MZ10F, Leica, Germany).

To investigate the anti-infection ability of Ti-PAA-NCl, peri-implantitis model was built after 4 weeks of osseointegration. After gingiva above implants was cut, non-absorbable 7^#^ silk ligatures were tied firmly around the neck of implants, pressed into gingival groove along the apical direction and left an end in the oral cavity to induce bacteria^54^. Oral cleaning was stopped, and ligatures were replaced every 2 weeks and finally removed after 8 weeks. Then, implants underwent a re-osseointegration progress to detect the long-lasting antibacterial effect against peri-implantitis. After 4 weeks, rabbits were euthanized for micro-CT scanning and implant–bone bonding force measurement, taking the situation of 4-week osseointegration and 8-week peri-implantitis as comparisons.

After the mandibulars were removed and fixed in 4% paraformaldehyde for 48 h, implants were scanned by micro-CT (SkyScan 1276, Bruker, Germany) with 100 kV in voltage and 200 µA in current. The region of interest was decided as a ring whose radius was 0.15 mm from the implant surface. Subsequently, 2D images were obtained by the Dataviewer software (v.1.5.6.2, Bruker, Karlsruhe, Germany) to determine the bone height by the vertical distance between the implant tip and the marginal bone level below the implant shoulder. 3D reconstructions were made by CTvox software (v.3.3.0, Bruker, Karlsruhe, Germany) to quantitatively analyse the ratio of BV/TV.

After the micro-CT scanning measurements, specimens were then used to measure the implant–bone bonding strength. The removal torques were tested using digital torque metre (HDP-5, ELET, China), and the maximum torque value was recorded as the torsion required for fracture of osseointegration interface.

### Assessment of long-lasting and renewable in vivo antibacterial effect against human bacterial colonies

The experimental process is approved by Ethics Committee of Hospital of Stomatology, Sun Yat-sen University (No. KQEC-2020-39-02). After signing informed consents, eight volunteers were selected for experiments that lasted for 4 weeks and three volunteers for 12 weeks. For each volunteer, two titanium disks (3 mm in diameter and 0.2 mm in thickness) with Ti-PAA-NCl surfaces were bonded on the middle-third of buccal surfaces of upper and lower first molars, respectively, on one side (right or left) based on randomization, while two titanium disks (3 mm in diameter and 0.2 mm in thickness) with Ti-OH surfaces were bonded on the same sites of the opposite side as the control group. Volunteers with these four disks continued their daily lives as usual, such as eating, tooth brushing and so on. To evaluate the long-lasting antibacterial and anti-biofilm effect, for each volunteer, one Ti-OH disk and one Ti-PAA-NCl disk were taken out after 4 or 12 weeks, and stained with LIVE/DEAD *Bac*Light Bacterial Viability Kit, followed by confocal observation of 2D and 3D morphologies. The covering area and fluorescence intensity of bacteria were quantitively analysed using the Image J software. On the other hand, to estimate the renewable antibacterial effect, at both 4-week and 12-week time points, the remaining disks (i.e. one Ti-OH disk and one Ti-PAA-NCl disk) in the mouth were irrigated with NaOCl solution (5%, pH = 7) for 15 min and pure water for another 5 min with rubber dam technique, and were taken out after 48 h to do the same detections.

### Statistical analysis

Data were expressed as the mean ± standard deviation from at least three parallel experiments. Statistical analysis was evaluated by SPSS 20.0 (IBM Corp., Chicago, USA) with Student’s *t* test, one-way analysis of variance (ANOVA) followed by Bonferroni multiple comparisons for data with homogeneity of variance, and Wilcoxon’s nonparametric test for data with heterogeneity of variance. *P* < 0.05 was considered statistically significant.

### Reporting summary

Further information on research design is available in the Nature Research Reporting Summary linked to this article.

## Supplementary information

Supplementary Information

Reporting Summary

## Data Availability

The authors declare that all the data supporting the findings of this study are available within the article and its Supplementary information or from the corresponding authors upon reasonable request.
