# Supplementary material for: Long-lasting renewable antibacterial porous polymeric coatings enable titanium biomaterials to prevent and treat peri-implant infection
Source: Nat Commun. 2021 Jun 3;12:3303. doi: 10.1038/s41467-021-23069-0 (PMC8175680; doi:10.1038/s41467-021-23069-0)
Supplement: Supplementary file 1 — Supplementary Information [file 41467_2021_23069_MOESM1_ESM.pdf]

Supplementary Information

Long-lasting Renewable Antibacterial Porous Polymeric Coatings Enable Titanium Biomaterials to Prevent and Treat Peri-implant Infection

Shuyi Wu[1,†], Jianmeng Xu[1,†], Leiyan Zou[1,†], Shulu Luo[1], Run Yao[1], Bingna Zheng[2], Guobin Liang[1], Dingcai Wu[2]*& Yan Li[1]*

[1] Department of Prosthodontics, Hospital of Stomatology, Guanghua School of Stomatology, Guangdong Provincial Key Laboratory of Stomatology, Sun Yat-sen University, Guangzhou 510055, P. R. China.

[2] Materials Science Institute, PCFM Lab and GDHPRC Lab, School of Chemistry, Sun Yat-sen University, Guangzhou 510275, P.R. China.

[†] Shuyi Wu, Jianmeng Xu and Leiyan Zou contributed equally to this work.

* Correspondence and requests for materials should be addressed to D.W. (email: wudc@mail.sysu.edu.cn) and Y.L. (email: liy8@mail.sysu.edu.cn).

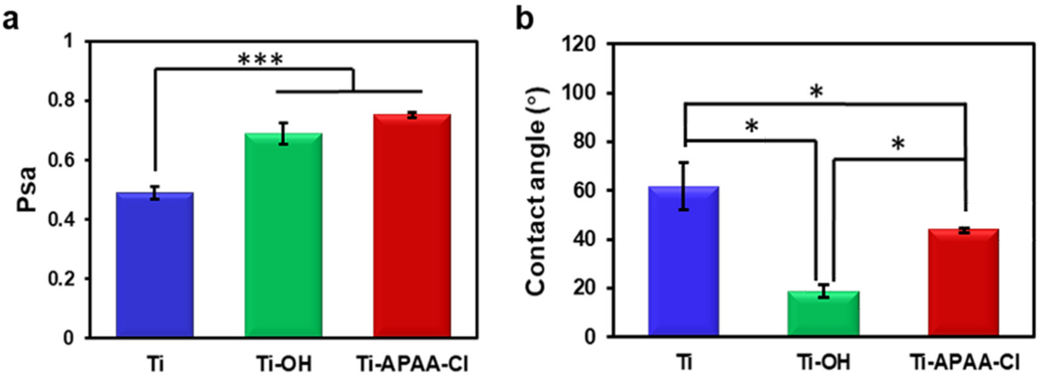

Supplementary Fig. 1 Roughness and water contact angle of Ti, Ti-OH and Ti-PAA-NCl. a Quantitative analysis of roughness (*n* = 3, *F* = 61.626 and *P* < 0.001 in one-way ANOVA, *** indicates *P* < 0.001 in Bonffereoni test). **b** Quantitative analysis of water contact angle (*n* = 3; * indicates *P* < 0.05, Wilcoxon nonparametric test). All error bars = s.d.

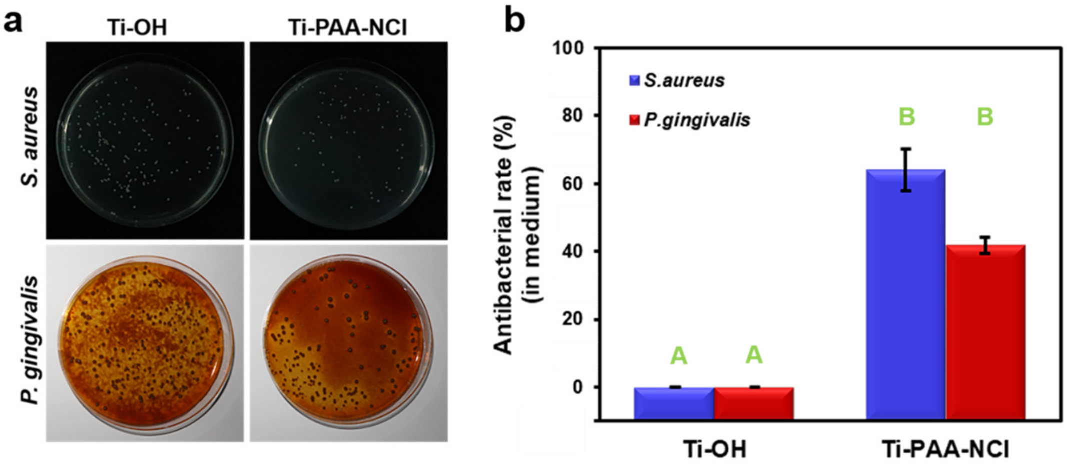

Supplementary Fig. 2 a Images of bacterial colonies formed by *S. aureus* and *P. gingivalis* from culture medium treated with Ti-OH and Ti-PAA-NCl. **b** Antibacterial rates in medium against *S. aureus* and *P. gingivalis* treated with Ti-OH and Ti-PAA-NCl. Significant differences between Ti-OH and Ti-PAA-NCl are marked by different letters (*n* = 3; *P* < 0.01; Student's t test). Error bars = s.d.

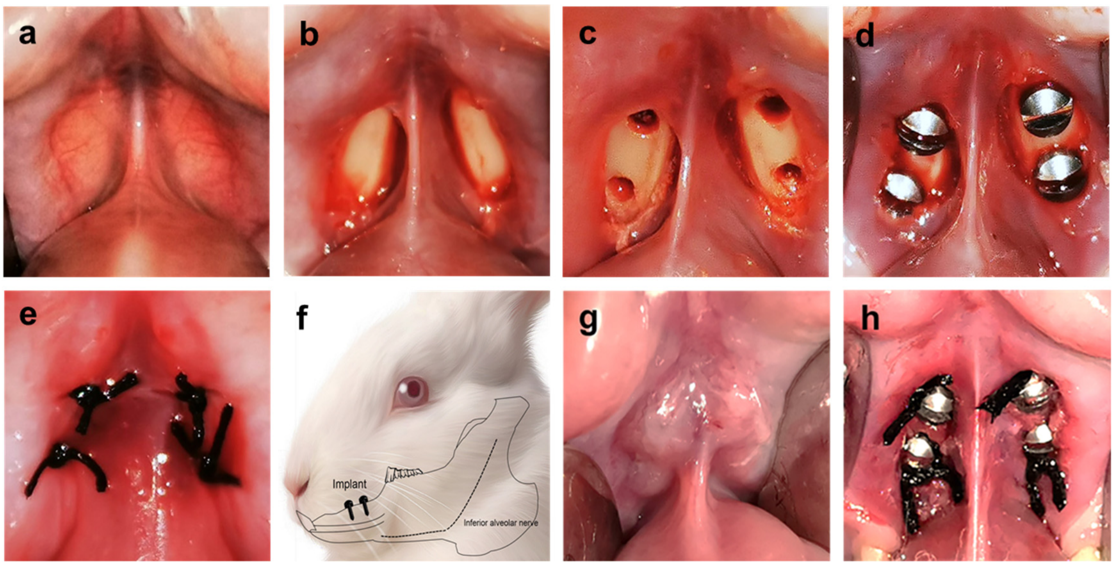

Supplementary Fig. 3 Processes of *in vivo* animal experiment. a Rabbit's bilateral edentulous areas of mandible. **b** Incisions and exposures of implant sites. **c** Holes drilled for mini-implants. **d** Implantation of mini-implants. **e** Closing the incisions by suturing. **f** Schematic diagram of implantation positions. **g** Healing of implant sites after 4 weeks. **h** Silk ligatures placed around the neck of implants with ends left in oral cavity to introduce bacteria.

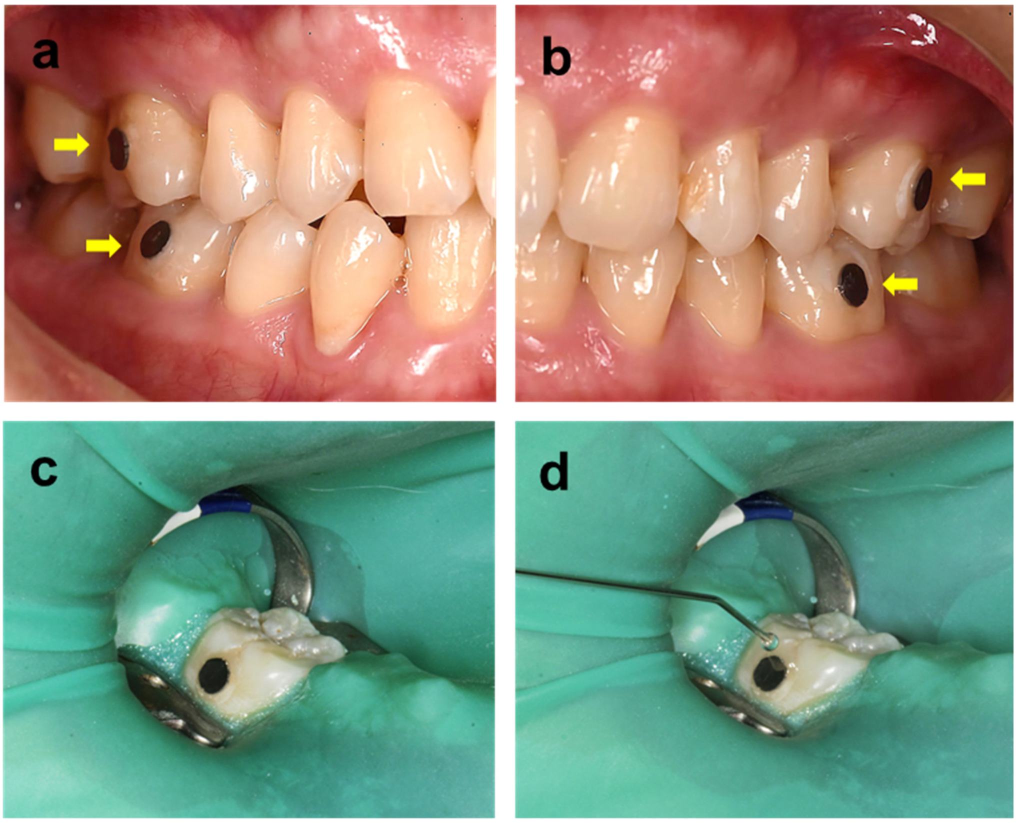

Supplementary Fig. 4 Processes of human intraoral experiment. a, b Bonding sites of tiny titanium disks (yellow arrows) on buccal surfaces of upper and lower first molars in volunteers' mouth. **c** Regional exposure of a tiny titanium disk with rubber dam and gum sealant for preparation of rechlorination. **d** Rechlorinating the coating by irrigating with NaOCl solution (5%, pH=7) for 15 min.

Supplementary Table 1 Inclusion criteria for patients with peri-implantitis

SN	Inclusion criteria
1	Obvious radiological evidence for vertical destruction of crestal bone
2	Vertical bone destruction associated with peri-implant pocket
3	Bleeding after gentle probing with blunt instrument
4	Tissues might or might not be swollen

Supplementary Table 2 Primer sequences used for RT-qPCR

Genes	Primers
OCN	Forward: 5’-GGACCATCTTTCTGCTCACTCTG-3’
	Reversed: 5’-TTCACTACCTTATTGCCCTCCTG-3’
OPN	Forward: 5’- CTTTCACTCCAATCGTCCCTAC-3’
	Reversed: 5’- CCTTAGACTCACCGCTCTTCAT-3’
RUNX2	Forward: 5’- AAGTGCGGTGCAAACTTTCT-3’
	Reversed: 5’- TCTCGGTGGCTGGTAGTGA-3’
GAPDH	Forward: 5’- TCGGTGTGAACGGATTTGG-3’
	Reversed: 5’- TCTCCACTTTGCCACTGCA-3’
